# Efficacy and non-target impact of spinosad, Bti and temephos larvicides for control of *Anopheles* spp. in an endemic malaria region of southern Mexico

**DOI:** 10.1186/1756-3305-7-55

**Published:** 2014-01-30

**Authors:** Carlos F Marina, J Guillermo Bond, José Muñoz, Javier Valle, Rodolfo Novelo-Gutiérrez, Trevor Williams

**Affiliations:** 1 Centro Regional de Investigación en Salud Pública - INSP, Tapachula, Chiapas 30700, Mexico; 2 ECOSUR, Tapachula, Chiapas 30700, Mexico; 3 Instituto de Ecología AC, Xalapa, Veracruz 91070, Mexico

## Abstract

**Background:**

The larvicidal efficacy of the naturally derived insecticide spinosad, for control of immature stages of *Anopheles albimanus* and associated culicids, was compared to that of synthetic and biological larvicides. Effects on non-target insects were also determined.

**Methods:**

A field trial was performed in replicated temporary pools during the rainy season, in southern Mexico. Pools were treated with 10 ppm a.i. spinosad (Tracer 480SC), Bti granules applied at 2 kg/ha (VectoBac WDG, ABG-6511), and 100 ml/ha temephos (50 EC), or an untreated control. Numbers of immature mosquitoes, and aquatic insects in pools were monitored for 20 weeks.

**Results:**

Samples of immature mosquitoes comprised approximately 10% *An. albimanus*, 70% *Culex* spp. (mostly *Cx. melanoconion* and *Cx. coronator*) and 20% *Uranotaenia lowii*. The most effective larvicides were spinosad and temephos that eliminated *An. albimanus* in 16 out of 20 post-treatment samples, or 9 weeks of continuous control of immature stages, respectively. These larvicides resulted in 15 and 5 weeks of elimination of *Culex* spp., respectively, or 20 and 4 weeks of continuous elimination of *U. lowii*, respectively. Bti treatment provided little consistent control. Aquatic insects were recorded comprising 3 orders, 20 families, 40 genera and 44 species. Shannon diversity index values (H’) for aquatic insects were highest in the control (0.997) and Bti (0.974) treatments, intermediate in the spinosad treatment (0.638) and lowest in the temephos treatment (0.520). Severely affected non-target insects in the spinosad and temephos treated pools were predatory Coleoptera, Hemiptera and Odonata, which in the case of spinosad was likely due to the high concentration applied. Bti had little effect on aquatic insects.

**Conclusions:**

The spinosad treatment retained larvicidal activity for markedly longer than expected. Spinosad is likely to be an effective tool for control of anopheline and other pool-breeding mosquitoes in tropical regions. Non-target effects of spinosad on aquatic insects merit further study, but were likely related to the concentration of the product used.

## Background

Temporary freshwater pools are island habitats that vary widely in the diversity of their macroinvertebrate fauna. Multiple physical factors influence the use of these ephemeral habitats by invertebrates, principally pool surface area, depth, permanence (hydroperiod), temperature, and salinity [1]. Similarly, biotic factors including predation, competition and the structure of food webs often determine the composition and abundance of aquatic invertebrate communities [2]. The ecological value of these habitats in the conservation of endangered biota, and concerns over the marked reduction in their abundance due to land drainage and changes in land use, are frequently at odds with their public health importance as breeding sites for medically important organisms, particularly mosquito vectors of human disease [3].

Many species of anopheline and culicid mosquitoes exploit temporary pools as oviposition sites across a diversity of natural, agricultural and urban environments. The rapid warming of water temperatures in pools and the presence of organic matter, such as leaf litter or algae, provide conditions suitable for the rapid development of mosquito immature stages [4]. Such pools also attract a number of mosquito predators, including amphibians, insects and crustaceans for which immature mosquitoes can be an important component of their diet. However, to improve the likelihood of offspring survival, mosquitoes tend to avoid oviposition in pools that contain predators [5], a process that appears to involve chemically-mediated detection of predator-related kairomones [6].

In Mexico, an extensive region of endemic malaria transmission has been reduced to two principal foci following several decades of State and Federal government programs targeted at eliminating *Anopheles* spp. breeding sites, where possible, by drainage or changes in land use. The foci of high malaria risk by transmission of *Plasmodium vivax* are restricted to the southern state of Chiapas that borders Guatemala, and a northern focus in the states of Chihuahua and Sinaloa that involves about 70% fewer cases than the Chiapas focus [7]. In Chiapas, the most important vectors of *P. vivax* are *Anopheles pseudopunctipennis* in the coffee-growing foothills region, and *Anopheles albimanus* along the lowland costal plain where livestock are grazed and mangoes or bananas are grown.

Vector control programs targeted at *Anopheles* spp. have been complemented by the application of larvicides to temporary pools that form in the rainy season, including DDT, malathion and temephos. Whereas the use of DDT and malathion has been discontinued, the organophosphate temephos (Abate) continues to be used widely. Five other compounds have received government approval for use in vector control in natural bodies of surface water in Mexico, namely the bacterial insecticide *Bacillus thuringiensis israelensis* (Bti), the naturally-derived insecticide spinosad, two insect growth regulators, novaluron and methoprene, and ethoxylated alcohols for production of surface monolayers. Due to their cost, these compounds are rarely used and temephos remains the compound of choice for larviciding of *Anopheles* breeding sites, despite concerns about the incidence of organophosphate resistance in *Anopheles* populations in the region [8-11].

In previous studies we reported that spinosad at concentrations between 1 and 10 parts per million (ppm) was a highly effective larvicide against *Aedes aegypti*, *Aedes albopictus* and *Culex* spp. that develop in water containers or abandoned car tires in urban or peri-urban habitats in Mexico [12-15]. These findings have been substantiated and expanded upon by others that have studied container and pool dwelling mosquito species across different parts of the world [16-22].

In the present study we compared the efficacy of spinosad and two other larvicides, temephos and Bti, for control of *An. albimanus* and associated culicids in temporary pools during the rainy season in the high-risk malaria region of Chiapas, Mexico. We also addressed the issue of the impact of these products on aquatic insect diversity, including species that predate immature mosquitoes, in the temporary pools. This represents the first study on use of spinosad for control of *Anopheles* spp. in an endemic malarial region of Latin America, and the first study on the effects of spinosad on the diversity of non-target aquatic insects in any country.

## Methods

### Insecticides

Spinosad was obtained as a suspension concentrate (Tracer 480SC, Dow AgroSciences LLC, Indiana, USA) containing 480 g active ingredient (a.i.)/l, which was the only commercial formulation of spinosad available in Mexico at the moment of the trial, and which had been used successfully for its larvicidal properties in previous studies [12-15]. Bti was obtained as a water dispersible granular formulation (VectoBac WDG, product code ABG-6511; Valent BioSciences Corp., Illinois, USA) containing 3,000 international toxicity units (ITU)/mg and 37.4% a.i. by weight. Finally, temephos (Abate) was obtained as a generic emulsifiable concentrate formulation containing 50% a.i. that is used by Mexico’s Secretaria de Salud for treatment of temporary pools that are oviposition sites for *Anopheles* spp. of public health importance in Mexico.

### Field experiment

A field experiment was performed in a coastal site at an altitude of 13 m above sea level, 30 m from the estuary of the River Coatán and 700 m from the Pacific Ocean beach at San Simón, in the municipality of Mazatán, Chiapas State, in southern Mexico (14°48′ N; 92°30′ W). This area of Chiapas is presently classified as a region of highest risk for transmission of malaria (*P. vivax*) in Mexico [7].

The experimental site was a flat uncultivated piece of land in which 16 small artificial ponds (four ponds per treatment) were dug over an area of 20 × 30 m. Each pond was 1.5 × 1.5 m (2.25 m^2^ in area) and 0.8 m deep. Each pond was lined with a transparent sheet of plastic and was filled to a depth of approximately 40 cm (mean ± SE: 38.4 ± 1.4 cm) with river water in mid-August 2008. Ponds were checked weekly for the presence of mosquito larvae. When larvae were observed to be present in all experimental ponds, the experiment began with weekly pre-treatment sampling beginning on 09 September 2008 and continued for 5 weeks until 06 October 2008. The following day (07 October), one of the following treatments was assigned at random to each pond: (i) 10 ppm a.i. spinosad (an average of 16.2 ml/pond of Tracer 480SC, based on the estimated volume of water in each pond [mean ± SD: 776 ± 169 liters; range: 630 – 990 liters]), ii) 25 μl of temephos 50% EC liquid, equivalent to 110 ml product/ha based on pond surface area, according to government recommended rates [11], iii) 450 mg VectoBac WDG water dispersible granules (product code ABG-6511, equivalent to the recommended rate of 2 kg granules/ha), (iv) control consisting of untreated water. All treatments were applied by mixing each product with approximately 1 liter of pond water in a plastic container prior to pouring each solution or suspension into the corresponding pond.

Ponds were monitored weekly for mosquito aquatic stages and for the presence of other aquatic insects during 15 weeks post-treatment and a final sample was taken at 20 weeks post-treatment. Prior to sampling, air temperature and water temperature were measured between 09.00 and 11.00 h using a digital thermometer. Water depth in each pond was measured using a graduated rule. Ponds were only supplemented with natural rainfall during the experimental period; no additional water was added. All insects were collected using cone-shaped nets, with an orifice diameter of 25 cm and a pore size of 0.7 × 0.17 mm (57 pores/cm^2^). Two samples were taken from each pond: a perimeter sample was taken by dragging the net around the edge of the pond, whereas a central sample was taken by taking an X-shaped sample from corner-to-corner of each pond. Immature mosquitoes and other aquatic insects were then placed in plastic trays, counted, identified, and registered according to genus, and returned to the pond from which they were taken so that sampling did not affect mosquito or aquatic insect populations.

A small sub-sample of mosquito larvae and pupae was taken from each pond and placed in 100 ml of water in a plastic bag for laboratory rearing and identification. A sub-sample of aquatic insects was taken by placing insects in 80% alcohol in vials, which were subsequently transported to the laboratory in insulated boxes for identification. At the end of the experiment plastic sheets were removed and the ponds were filled with soil to avoid them becoming long-term breeding sites for *Anopheles* spp.

Field collected samples of mosquito larvae and pupae were reared in enamel trays in the laboratory insectary at 28 ± 2°C, using a ground rabbit food diet. Mosquitoes were identified to species following adult emergence. Aquatic insects were identified to genus and species using published keys. All identified insects were registered in the Mexican Council on Biodiversity (CONABIO) database [23].

### Statistical analyses

Due to the low numbers of mosquito larvae and pupae in some treatments, records of *Anopheles* spp., *Culex* spp. and *Uranotaenia* spp. were pooled at each sample time, normalized by ln(*x* + 1) transformation, and used in a mixed model analysis with a Toeplitz correlation structure specified in SAS v. 8.5 (SAS Institute Inc., Cary, NC). To determine the significance of treatments, multiple comparisons were performed based on a Bonferroni adjusted critical α value of 0.005.

To validate the sampling effort directed at aquatic insects, species accumulation curves were compared by fitting quadratic regressions. The slopes of each regression were then compared by analysis of covariance with mean separation by Tukey test (α = 0.05). Aquatic insect diversity was estimated by calculation of Shannon index (H’) values. The precision of H’ values was estimated by jack-knife procedures [24].

## Results

### Pool characteristics and sampling of immature mosquitoes

Average (±SE) air temperature during sampling was 29.3 ± 0.1°C (range 25 – 34°C), whereas average pond water temperature was 26.9 ± 0.3°C (range 23 – 32°C). Average water depth was 31.3 ± 2.8 cm during the experimental period, although water depth tended to decrease during the experiment in all treatments (Additional file 1: Figure S1). In the final sample, taken at 20 weeks post-treatment, two pools in the spinosad treatment and one pool in the control had dried up and were not included in the results.

During the 5 week pre-treatment sampling period, a total of 6,904 larvae and pupae were recorded belonging to the genera *Anopheles*, *Culex* and *Uranotaenia* (Table 1). The most abundant of these genera were *Culex* spp. that represented 75.0% of recorded individuals, followed by *Uranotaenia* (14.9%) and *Anopheles* spp. (10.1%)

**Table 1:** Summary of larvae and pupae sampled in temporary pools pre- and post-treatment with larvicides at San Simón beach, Mazatán municipality, Chiapas, Mexico

**Genera**				
**Treatment**	***Anopheles *** **spp.**	***Culex *** **spp.**	***Uranotaenia *** **spp.**	**Total**
Pre-treatment	698	5,180	1,026	6,904
Post-treatment				
Control	288	5,242	1,975	7,505
Bti	527	2,495	406	3,428
Spinosad	31	8	0	39
Temephos	241	3,716	1,401	5,358
Totals:	1,087	11,461	3,782	16,330

During the 20 weeks of post-treatment sampling a total of 16,330 larvae + pupae of these three genera were recorded (Table 1). Overall, the highest incidence of culicid larvae and pupae was observed in the control treatment (46.0% of total culicids recorded), followed by the temephos treatment (32.8%), the Bti treatment (21.0%) and finally the spinosad treatment (0.2%). The relative abundance of each genus was generally similar to that of pre-treatment sampling; *Culex* spp. comprised 70.2% of recorded larvae + pupae, followed by *Uranotaenia* spp. (23.2%) and *Anopheles* spp. (6.6%).

### Identification of laboratory reared samples

A total of 693 individuals were identified from the adults that emerged following laboratory rearing of field collected larvae + pupae. Of the 68 individuals of *Anopheles* that emerged, 97% (29♀, 39♂) were identified as *An. albimanus* and the remaining two individuals were *An. punctimacula*. Of the 567 adults of *Culex*, 56.6% (N = 321; 180♀, 141♂) were identified as *Culex melanoconion*, 36.3% were *Culex coronator* (N = 206; 103♀, 103♂), 2.7% were *Culex nigripalpus* (N = 15; 8♀, 7♂), 2.1% were *Culex quinquefasciatus* (N = 12; 7♀, 5♂), and the remaining 13 individuals could only be identified to genus. All 53 of the *Uranotaenia* adults were identified as *Uranotaenia lowii* (N = 53; 32♀, 21♂). Finally, three individuals of the genus *Limatus* emerged, but could not be identified to species.

### Efficacy of larvicides against *Anopheles* spp

Larvae and pupae of *Anopheles* spp. were recorded in all ponds at most time points during pre-treatment sampling, with averages of 0.0 – 31.8 larvae + pupae/pond during this 5 week period (Table 2). Average numbers of *Anopheles* spp. larvae + pupae in the control pools varied from 0.0 – 20.5 larvae + pupae/pool during the 20 weeks of post-treatment sampling. Larvicide treatments resulted in a significant reduction in numbers of *Anopheles* larvae + pupae during the post-treatment period compared to the control (F_20,233_ = 8.47, *P* < 0.001). The most effective larvicide treatment was spinosad, which eliminated *Anopheles* larvae + pupae in 16 out of 20 samples, although lower numbers were recorded on weeks 6, 7, 12 and 20 in this treatment (Table 2). Temephos also provided continuous effective control of larvae + pupae for 9 weeks post-treatment. In contrast, Bti treatment did not result in a significant reduction in numbers of *Anopheles* immatures compared to the control, except for one sample taken at 12 weeks post-treatment.

**Table 2:** **Mean (±SE) numbers of *Anopheles *** **spp. larvae and pupae sampled pre- and post-treatment with larvicides in temporary pools at San Simón beach, Mazatán municipality, Chiapas, Mexico**

	**Treatment**			
**Time (Weeks)**	**Control**	**Bti**	**Spinosad**	**Temephos**
Pre-treatment				
-5	0.0 ± 0.0^a^	4.8 ± 2.3^a^	2.5 ± 2.2^a^	2.3 ± 2.3^a^
-4	0.0 ± 0.0^b^	0.0 ± 0.0^b^	2.3 ± 1.9^ab^	5.0 ± 4.4^a^
-3	9.5 ± 8.2^a^	18.0 ± 12.8^a^	12.3 ± 10.0^a^	15.8 ± 15.1^a^
-2	1.3 ± 0.8^a^	11.3 ± 6.0^a^	4.0 ± 1.7^a^	1.0 ± 1.0^a^
-1	8.0 ± 5.7^a^	19.8 ± 5.1^a^	25.3 ± 14.9^a^	31.8 ± 22.2^a^
Post-treatment				
1	1.3 ± 0.8^a^	5.5 ± 5.5^a^	0.0 ± 0.0^a^	0.0 ± 0.0^a^
2	0.8 ± 0.8^b^	9.8 ± 5.5^a^	0.0 ± 0.0^b^	0.0 ± 0.0^b^
3	2.5 ± 2.5^b^	13.0 ± 5.6^a^	0.0 ± 0.0^b^	0.0 ± 0.0^b^
4	3.3 ± 3.3^ab^	9.3 ± 6.6^a^	0.0 ± 0.0^b^	0.0 ± 0.0^b^
5	4.8 ± 4.1^a^	1.5 ± 0.6^ab^	0.0 ± 0.0^b^	0.0 ± 0.0^b^
6	0.0 ± 0.0^b^	4.0 ± 1.8^a^	4.5 ± 4.5^a^	0.0 ± 0.0^b^
7	1.5 ± 1.5^a^	0.8 ± 0.8^a^	1.5 ± 1.5^a^	0.0 ± 0.0^a^
8	0.0 ± 0.0^b^	4.0 ± 1.4^a^	0.0 ± 0.0^b^	0.0 ± 0.0^b^
9	1.5 ± 1.2^a^	1.5 ± 1.0^a^	0.0 ± 0.0^a^	0.0 ± 0.0^a^
10	7.5 ± 5.7^a^	7.5 ± 5.2^a^	0.0 ± 0.0^b^	2.3 ± 2.3^a^
11	2.5 ± 1.0^a^	4.0 ± 3.4^a^	0.0 ± 0.0^b^	11.5 ± 9.3^a^
12	5.5 ± 4.6^a^	0.3 ± 0.3^b^	0.3 ± 0.3^b^	0.0 ± 0.0^b^
13	11.8 ± 7.9^a^	27.3 ± 7.5^a^	0.0 ± 0.0^b^	21.5 ± 20.2^a^
14	7.5 ± 5.7^a^	6.8 ± 4.2^a^	0.0 ± 0.0^b^	0.8 ± 0.8^b^
15	11.8 ± 3.9^a^	10.3 ± 2.5^a^	0.0 ± 0.0^b^	8.0 ± 3.4^a^
20	20.5 ± 3.5^a^	26.5 ± 19.3^a^	2.0 ± 2.0^b^	16.3 ± 4.5^a^

Values followed by identical letters did not differ significantly for comparisons within rows (mixed model analysis on ln (*x* + 1) transformed values with Toeplitz correlation structure, P > 0.005 following Bonferroni correction).

### Efficacy of larvicides against *Culex* spp

Pre-treatment sampling revealed high numbers of *Culex* spp. larvae + pupae in untreated ponds that varied from an average of 3.5 – 185.5 larvae + pupae/pool (Table 3). Larvicide treatments resulted in a significant reduction in numbers of *Culex* spp. compared to the control (F_20,233_ = 13.95, *P* < 0.001). Spinosad treatment provided absolute control of *Culex* spp. larvae + pupae for a continuous period of 15 weeks. Temephos treatment resulted in absolute control of *Culex* spp. for a continuous period of 5 weeks, after which numbers of larvae + pupae were reduced compared to the control for an additional 3 weeks (Table 3). In contrast, Bti treatment resulted in a reduction in *Culex* spp. compared to the control in samples taken at intermittent time points: 1, 3, 6, 7 and 8 weeks post-treatment.

**Table 3:** **Mean (±SE) numbers of *Culex *** **spp. larvae and pupae sampled pre- and post-treatment with larvicides in temporary pools at San Simón beach, Mazatán municipality, Chiapas, Mexico**

	**Treatment**			
**Time (Weeks)**	**Control**	**Bti**	**Spinosad**	**Temephos**
Pre-treatment				
-5	8.5 ± 3.9^b^	26.0 ± 12.7^a^	49.5 ± 26.6^a^	5.7 ± 2.9^b^
-4	19.8 ± 12.6^b^	44.0 ± 30.6^ab^	105.8 ± 56.2^a^	3.5 ± 1.3^bc^
-3	56.0 ± 22.8^a^	18.0 ± 7.0^a^	185.5 ± 129.8^a^	71.3 ± 61.9^a^
-2	65.0 ± 45.9^b^	74.3 ± 45.9^ab^	125.8 ± 73.1^a^	17.3 ± 6.0^bc^
-1	122.3 ± 43.7^a^	135.3 ± 84.3^a^	125.3 ± 67.6^a^	36.5 ± 7.6^a^
Post-treatment				
1	132.3 ± 68.8^a^	2.8 ± 2.4^b^	0.0 ± 0.0^b^	0.0 ± 0.0^b^
2	21.0 ± 7.6^a^	15.5 ± 12.9^a^	0.0 ± 0.0^b^	0.0 ± 0.0^b^
3	143.0 ± 70.7^a^	51.5 ± 35.8^b^	0.0 ± 0.0^c^	0.0 ± 0.0^c^
4	90.5 ± 48.4^a^	45.5 ± 36.3^a^	0.0 ± 0.0^b^	0.0 ± 0.0^b^
5	49.8 ± 30.8^a^	13.0 ± 7.3^a^	0.0 ± 0.0^b^	0.0 ± 0.0^b^
6	184.8 ± 94.4^a^	30.5 ± 29.5^b^	0.0 ± 0.0^c^	0.8 ± 0.5^bc^
7	238.5 ± 133.2^a^	19.5 ± 14.2^b^	0.0 ± 0.0^c^	0.8 ± 0.8^c^
8	82.8 ± 35.8^a^	8.8 ± 6.9^b^	0.0 ± 0.0^c^	6.8 ± 4.5^b^
9	40.0 ± 19.5^a^	26.5 ± 18.5^a^	0.0 ± 0.0^b^	7.8 ± 5.7^ab^
10	55.0 ± 25.0^a^	28.8 ± 17.0^a^	0.0 ± 0.0^b^	19.3 ± 9.9^a^
11	39.0 ± 15.5^a^	23.5 ± 17.5^ab^	0.0 ± 0.0^c^	47.5 ± 16.8^a^
12	54.8 ± 17.3^a^	28.0 ± 17.2^a^	0.0 ± 0.0^b^	54.3 ± 20.7^a^
13	85.8 ± 43.7^ab^	46.0 ± 34.2^b^	0.0 ± 0.0^c^	184.8 ± 86.5^a^
14	44.8 ± 29.6^b^	136.3 ± 112.9^b^	0.0 ± 0.0^c^	327.8 ± 116.4^a^
15	42.3 ± 25.4^b^	125.3 ± 76.8^a^	0.0 ± 0.0^c^	228.8 ± 77.4^a^
20	13.0 ± 11.0^ab^	22.5 ± 15.7^a^	2.7 ± 2.7^b^	50.8 ± 26.6^a^

Values followed by identical letters did not differ significantly for comparisons within rows (mixed model analysis on ln (*x* + 1) transformed values with Toeplitz correlation structure, P > 0.005 following Bonferroni correction).

### Efficacy of larvicides against *Uranotaenia* spp

Records of the presence of *Uranotaenia* spp. were only taken at -2 weeks and -1 week pre-treatment; during this period average numbers varied from 9.5 – 82.3 larvae + pupae/pool (Table 4). Larvicide treatments resulted in a significant reduction in *Uranotaenia* spp. numbers compared to that of the control treatment (F_17,197_ = 5.96, *P* < 0.001). Once again, the most effective treatment was that of spinosad, that resulted in elimination of *Uranotaenia* spp. larvae + pupae for a continuous period of 20 weeks post-treatment. Temephos treatment resulted in 4 weeks of continuous elimination of *Uranotaenia* spp. followed by 2 weeks of reduced numbers, after which numbers of *Uranotaenia* spp. were similar to that of the control treatment. In contrast, average numbers of larvae + pupae in the Bti treatment were reduced compared to the control in samples taken at 1, 6 and 10 weeks post-treatment (Table 4).

**Table 4:** **Mean (±SE) numbers of *Uranotaenia *** **spp. larvae and pupae sampled pre- and post-treatment with larvicides in temporary pools at San Simón beach, Mazatán municipality, Chiapas, Mexico**

	**Treatment**			
**Time (Weeks)**	**Control**	**Bti**	**Spinosad**	**Temephos**
Pre-treatment				
-2	14.5 ± 11.2^a^	31.5 ± 20.1^a^	33.5 ± 14.5^a^	9.5 ± 5.2^a^
-1	82.3 ± 43.2^a^	34.8 ± 15.4^a^	34.5 ± 33.2^a^	16.0 ± 7.2^a^
Post-treatment				
1	61.8 ± 28.6^a^	3.8 ± 1.7^b^	0.0 ± 0.0^b^	0.0 ± 0.0^b^
2	19.5 ± 14.6^a^	2.5 ± 1.9^ab^	0.0 ± 0.0^b^	0.0 ± 0.0^b^
3	65.5 ± 59.3^a^	7.0 ± 3.7^a^	0.0 ± 0.0^b^	0.0 ± 0.0^b^
4	35.3 ± 32.6^a^	1.5 ± 1.2^ab^	0.0 ± 0.0^b^	0.0 ± 0.0^b^
5	42.0 ± 24.4^a^	5.0 ± 2.9^ab^	0.0 ± 0.0^b^	0.3 ± 0.3^b^
6	78.3 ± 60.8^a^	1.3 ± 1.3^b^	0.0 ± 0.0^b^	1.3 ± 0.6^b^
7	18.8 ± 13.3^a^	1.5 ± 1.2^ab^	0.0 ± 0.0^b^	11.5 ± 7.3^a^
8	13.5 ± 7.8^a^	3.8 ± 1.5^ab^	0.0 ± 0.0^b^	28.0 ± 17.3^a^
9	28.5 ± 28.2^a^	3.0 ± 3.0^ab^	0.0 ± 0.0^b^	15.0 ± 7.7^a^
10	21.5 ± 17.7^a^	4.0 ± 2.3^b^	0.0 ± 0.0^b^	26.5 ± 18.2^a^
11	71.0 ± 71.0^a^	4.8 ± 2.3^ab^	0.0 ± 0.0^b^	46.3 ± 28.0^a^
12	10.5 ± 10.5^b^	10.8 ± 9.8^b^	0.0 ± 0.0^c^	41.8 ± 20.7^a^
13	13.5 ± 11.9^b^	12.3 ± 5.0^b^	0.0 ± 0.0^c^	58.0 ± 26.9^a^
14	7.3 ± 3.7^b^	17.8 ± 11.7^b^	0.0 ± 0.0^c^	74.0 ± 29.3^a^
15	5.3 ± 2.9^b^	19.8 ± 7.2^ab^	0.0 ± 0.0^c^	26.0 ± 9.0^a^
20	3.5 ± 2.5^ab^	3.0 ± 3.0^b^	0.0 ± 0.0^c^	21.8 ± 8.4^a^

Values followed by identical letters did not differ significantly for comparisons within rows (mixed model analysis on ln (*x* + 1) transformed values with Toeplitz correlation structure, P > 0.005 following Bonferroni correction).

### Taxa richness of other aquatic insects

**Figure 1:**
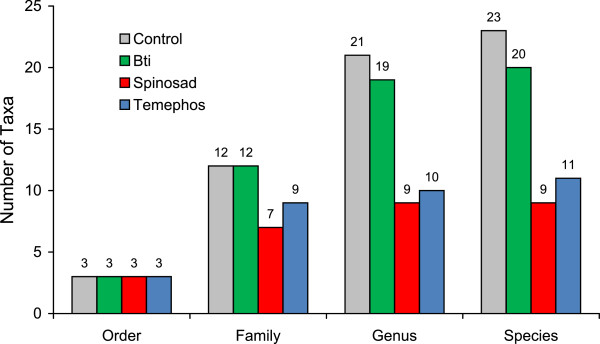
**Taxa richness of aquatic insects in experimental pools following treatment with one of three larvicides or an untreated control.** Columns indicate total numbers of orders, families, genera and species collected during the 20 week post-treatment period. Values above columns indicate the total numbers of taxa sampled.

A total of 19,741 aquatic insects were recorded comprising 3 orders, 20 families, 40 genera and 44 species. Overall, the most abundant order was Hemiptera (18,557 individuals from 11 families, 14 genera and 14 species), followed by Coleoptera (692 individuals from 4 families, 21 genera and 24 species). The least abundant order was Odonata (492 individuals from 5 families, 6 genera and 6 species). Pre-treatment sampling resulted in the collection of 5,462 individuals from 3 orders, 17 families, 30 genera and 33 species, whereas post-treatment sampling resulted in the identification of 14,279 individuals from 3 orders, 15 families, 26 genera, and 28 species. Comparison among treatments during the post-treatment period revealed that Bti treatment resulted in very minor changes in taxa richness involving two fewer genera and three fewer species compared to samples taken from control pools (Figure 1). In contrast, temephos and spinosad treatments both resulted in 3-5 fewer families recorded and ~50% reduction in the numbers of genera and species in pools treated with these insecticides.

**Figure 2:**
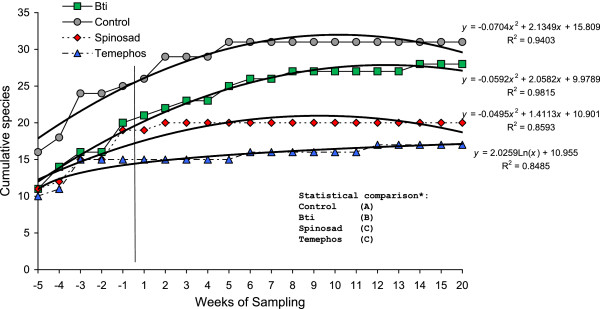
**Species accumulation curves for aquatic insect species from experimental pools in southern Mexico.** Pools were subjected to one of three larvicidal treatments or an untreated control. Curves were fitted to empirical results by quadratic or logarithmic regression. *Treatments followed by different letters differed significantly; analysis of covariance, Tukey test α = 0.05.

Species accumulation curves plotted for each of the four treatments revealed that in all treatments the curve plateaued, indicating that sampling effort was sufficient to estimate species richness in experimental pools (Figure 2). Analysis of the slopes of each fitted regression indicated that treatments differed significantly in terms of rate of accumulation of species: spinosad and temephos treatments were similar and both differed significantly from the control and Bti treatments, the latter two treatments also differed significantly from one another (F_3,76_ = 8.50, *P* <0.001).

### Aquatic insect diversity

**Figure 3:**
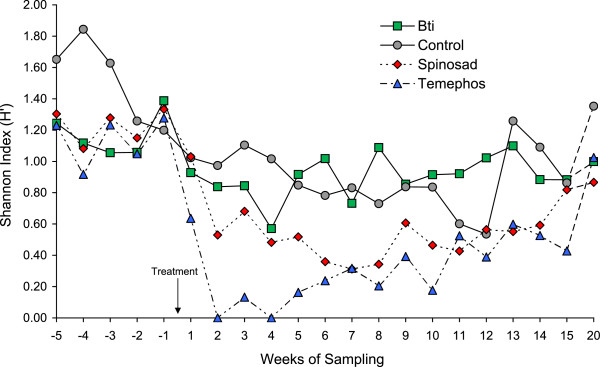
**Changes in Shannon diversity index values for experimental pools subjected to one of three larvicidal treatments and an untreated control.** Pre-treatment (5 weeks) and post-treatment (20 weeks) sampling values are shown.

Shannon diversity plots over time confirmed the tendency observed in the accumulation curve analysis. Pre-treatment values in each treatment varied between 0.92 and 1.84 depending on treatment and sample time (Figure 3). Application of temephos resulted in a marked reduction in diversity values to zero at 2 and 4 weeks post-treatment, that gradually returned to control pool values during the 20 week post-treatment sampling period. In contrast, Bti treatment resulted in a brief 4 week reduction in diversity index values that then fluctuated around control pool values for the remainder of the trial.

Total diversity values (H’) were highest in the control (0.997) and Bti (0.974) treatments, intermediate in the spinosad treatment and lowest in the temephos treatment (Table 5). Jack-knife estimates of the Shannon index values indicated that H’ values were highly accurate with a slight underestimate, in the range of 1.32 to 2.70%, depending on treatment. Confidence intervals for jack-knife estimates of H’ overlapped broadly between control and Bti treatments whereas no overlap in estimated confidence intervals were observed for the spinosad and temephos treatments compared to the control.

**Table 5:** Shannon index H’ values of aquatic insect diversity in temporary pools in San Simón, Mazatán, Chiapas, Mexico

**Treatment**	**H’ (All samples)**	**Jackknifed ϕ (± SE)**	**Confidence limits (95%)**		**Error (%)**	**Observations**
			**Lower**	**Upper**		
Control	0.997	1.014 ± 0.067	0.933	1.154	1.68	H’ was slightly underestimated
Bti	0.974	0.993 ± 0.066	0.848	1.068	1.93	H’ was slightly underestimated
Spinosad	0.638	0.646 ± 0.047	0.482	0.890	1.32	H’ was slightly underestimated
Temephos	0.520	0.534 ± 0.052	0.412	0.654	2.70	H’ was slightly underestimated

Pools were subjected to one of three larvicide treatments or an untreated control.

The most severely affected insect species in the spinosad-treated pools were the diving beetle *Laccophilus fasciatus* (Coleoptera: Dytiscidae), the backswimmer *Buenoa margaritacea* (Hemiptera: Notonectidae) and nymphs of the dragonfly *Anax amazili* (Odonata: Aeshnidae). These same species were also severely reduced in temephos treated pools, in addition to the water-measurer, *Hydrometra wileyae* (Hemiptera: Hydrometridae), and the water-strider, *Platyvelia brachialis* (Heteroptera: Veliidae) (data not shown).

## Discussion

In this field study, in an endemic malaria region of southern Mexico, a suspension concentrate formulation of spinosad was more effective than temephos or Bti as a larvicide for control of *An. albimanus*, *Culex* spp. and *Uranotaenia* spp. in artificial pools. Overall, the period of control in spinosad treated ponds was approximately twice that of temephos, whereas Bti provided no effective control at the rate tested. In this respect, the present study represents one of the first replicated studies on the efficacy of spinosad as an anopheline larvicide in the field. In a single previous study in Iran, treatment of 1 m^2^ artificial pools with 40 – 50 g/ha of spinosad granules or suspension concentrate resulted in ~100% mortality of *Anopheles culicifacies*, *Anopheles stephensi* and *Culex* spp. larvae for 9-11 days post-treatment [25].

Other studies on anopheline susceptibility to spinosad have focused on determining the concentration-mortality response in laboratory assays, and have identified *Anopheles gambiae* and *An. pseudopunctipennis* as the most susceptible species and *An. albimanus* and *An. stephensi* as the least susceptible species in toxicity assays, whereas *An. sinensis* was of intermediate susceptibility [26]. In contrast, field trials against *Culex* spp. have been more extensive and have consistently reported high larvicidal efficacy of spinosad against *Culex pipiens* in septic tanks in Turkey [16], *Culex pusillus* and *Cx. pipiens* in flooded fields in Egypt [27], *Culex quinquefasciatus* in drains and disused wells in India [18], and *Cx. pipiens pipiens* or *Culex restuans* in catch basins in Connecticut [21].

The concentration of spinosad (10 ppm) was at the upper limit of the range of concentrations tested by us in larvicide trials in this region (1-10 ppm); this was likely to have been highly influential in the excellent performance of this product as a larvicide in our experimental pools. We selected this concentration for testing as we expected rapid degradation of the product by photolysis in the sunny and relatively shallow pools used in this study [28]. Previous studies, at a distance of 24 km from the site of the present study, had estimated the half-life of spinosad in clean water in plastic trays exposed to direct sunlight and high temperatures at approximately 2.1 days [13]. We predicted that the concentration of 10 ppm spinosad would have fallen to below the laboratory LC_50_ value (0.02 ppm) within 3 weeks. As such, the trial was planned to last approximately 8 weeks, at which time we expected complete loss of larvicidal activity in the spinosad-treated pools. It was clear, however, that spinosad persisted and remained toxic to all three major mosquito genera, for at least 20 weeks post-application, which represents an important finding of the present study. As pools were located in sunny positions that are preferred by *An. albimanus*[29], water turbidity or shade within each pool provided by fallen leaf debris may have contributed to the greater than expected persistence of spinosad [26], although water turbidity or organic matter in pools were not monitored during the present experiment. In hindsight, it would have been useful to test lower concentrations, such as the 1 ppm and 5 ppm concentrations previously tested by us for control of *Ae. aegypti* and *An. albopictus* in containers or car tires [12-15]. In a previous trial, concentrations of 0.05 – 0.5 ppm spinosad resulted in over 95% control of *Culex* spp. larvae for a period of 7 – 35 days in outdoor pools or tubs in California [17]. Similarly, a recent tablet formulation (Natular TM, Clarke Mosquito Control Products Inc., Illinois), that is designed for use in water tanks and similar containers at a concentration of up to 1.6 ppm spinosad, was approved for use in Mexico in 2012.

The poor performance of Bti granules, which resulted in a brief reduction in numbers of *Culex* spp. and *Uranotaenia* spp., but no significant effect on *Anopheles* spp., agrees with previous findings on the larvicidal effects of this entomopathogen on *Aedes* spp. and *Culex* spp. [30,31]. Interestingly, the intermittent occurrence of reduced numbers of *Culex* spp. and *Uranotaenia* spp. observed in the Bti-treated pools suggests a low level of inoculum recycling. This phenomenon, involving the production and liberation of transmissible stages (spores and protoxin crystals) in the cadavers of pathogen-killed insects, can be responsible for periodic fluctuations in insect population densities, including mosquitoes [32]. Bti-based insecticides have been used successfully to control anophelines [30,31,33], but it appears that in the present study, control of *Anopheles* spp. requires a higher dose than that recommended by the manufacturer, or that the high intensity of solar radiation in the pools rapidly deactivated the protoxin crystals. The duration of larvicidal activity provided by Bti based products often depends on the location of the habitat [30], with greater efficacy and extended persistence of Bti treatments in shaded habitats [32,34], compared to those applied to habitats exposed to direct sunlight [35,36]. Moreover, the residual activity of Bti is not markedly improved by applying more product [35,37,38]. In contrast, products based on *Bacillus sphaericus* can have high activity against *Anopheles* spp. and are more frequently used against vectors from this genus than Bti [31,39].

The larvicidal activity of temephos was favorable for each of the three genera of mosquitoes. Temephos treatment provided 4-5 weeks of absolute control and an additional 2-3 weeks of partial control, i.e., numbers of larva + pupae that were significantly lower than in the control pools, for both *Culex* spp. and *Uranotaenia* spp. This compares to 10 weeks of absolute control of *Anopheles* spp. The low cost, high efficacy and low mammalian toxicity of this compound means that it is likely to be the first-choice larvicide for treating standing surface waters and water in tanks and other natural or man-made containers in developing countries, such as Mexico, that have limited resources available for public health programs. Previous studies carried out by us in southern Mexico revealed that a temephos granular mineral formulation provided between 4 and 10 weeks of absolute control of *Aedes* spp. and *Culex* spp. in cemetery flower vases or abandoned car tires [13-15], making it the most widely used larvicide in mosquito control programs in Mexico [11]. Organophosphate insecticides, particularly temephos and malathion, are also used as larvicides in many other tropical countries, although resistance has developed in some mosquito species [40,41].

Both spinosad and temephos treatments reduced species and genera richness of aquatic insects, whereas Bti had only minor effects on the numbers of species and genera, compared to the control pools, in line with previous studies on the high specificity of Bti-based products [42]. Similarly, aquatic insect diversity was significantly reduced in the spinosad and temephos treatments and took approximately 11-12 weeks to return to values observed in the control and Bti treatments (Figure 3). Species accumulation curves plateaued in all treatments indicating that sampling effort was sufficient for accurate estimation of aquatic insect diversity [43]. As the toxicity of any compound depends largely on the dose acquired by the exposed organism, it is important to note that the non-target effects observed in the spinosad-treated experimental pools probably reflected the comparatively high concentration of this product used in our study. Lower concentrations are likely to have a lesser effect on the non-target fauna given the selective ecotoxicological profile of spinosad [26]. Previous attempts to estimate the toxicity of spinosad to aquatic invertebrates identified Plecoptera as being sensitive to spinosad [44], and Ephemeroptera as being more sensitive than Odonata [45]. Predatory ostracods and *Toxorhynchites theobaldi* were eliminated or almost eliminated from spinosad-treated car tire habitats in southern Mexico [15], whereas a diversity of lethal and sublethal effects have been reported in *Daphnia* spp. and related cladocercans [46-48].

## Conclusions

We conclude that treatment of temporary breeding pools with spinosad is likely to prove to be a highly effective tool for control of anopheline vectors and other pool-breeding mosquitoes in tropical regions. The high efficacy and stability of this product in breeding pools may partially compensate for the higher cost of spinosad-based formulations compared to established larvicides, such as temephos. However, additional studies using lower application rates are required to determine the cost-effectiveness of spinosad-based mosquito control measures. Moreover, given its growing use as a larvicide in developed and developing countries, the issue of non-target effects of spinosad on aquatic insects merits detailed and systematic study.

## Competing interests

The authors declare that they have no competing interests.

## Authors’ contributions

CFM obtained funding via a competitive proposal. CFM, JGB, TW, JV designed the experiments. CFM, JGB, JM performed field studies. JM, CFM performed laboratory rearing and identification of mosquitoes. RNG, JGB identified aquatic insects. JV, CFM, JGB, TW performed statistical analyses. CFM, TW wrote the manuscript. All authors read and approved the final version of the manuscript.

## Supplementary Material

Additional file 1: Figure S1 Average water depth in experimental pools measured at moment of sampling during the period of the experiment. Pools were subjected to natural precipitation and water was not added by researchers at any time following construction and initial filling of the pools with water. At the final sample taken at 20 weeks post-treatment two pools in the spinosad treatment and one pool in the control treatment had dried up and were not included in the results. Vertical bars indicate SE. Click here for file
