# Supplementary figures and images for: Efficacy and non-target impact of spinosad, Bti and temephos larvicides for control of Anopheles spp. in an endemic malaria region of southern Mexico
Source: Parasit Vectors. 2014 Jan 30;7:55. doi: 10.1186/1756-3305-7-55 (PMC3915226; doi:10.1186/1756-3305-7-55)

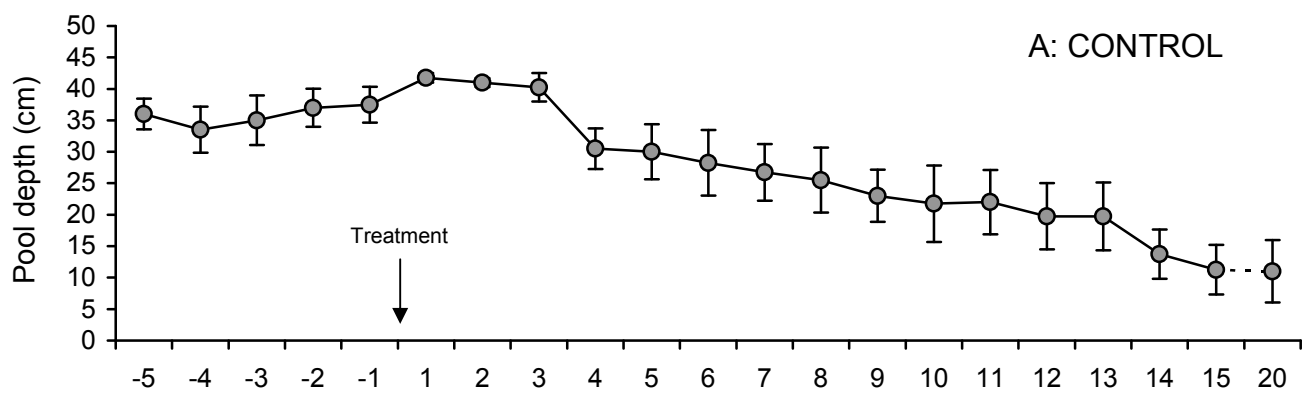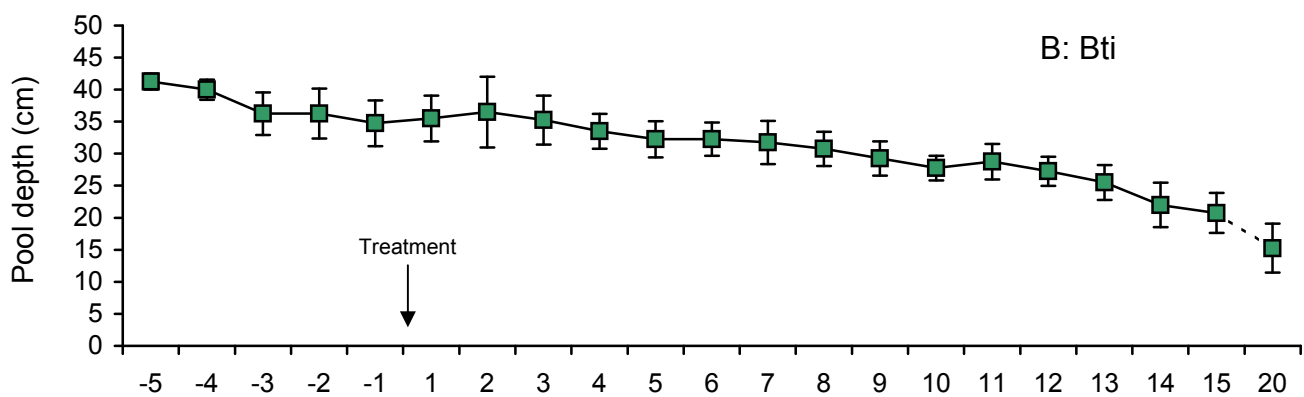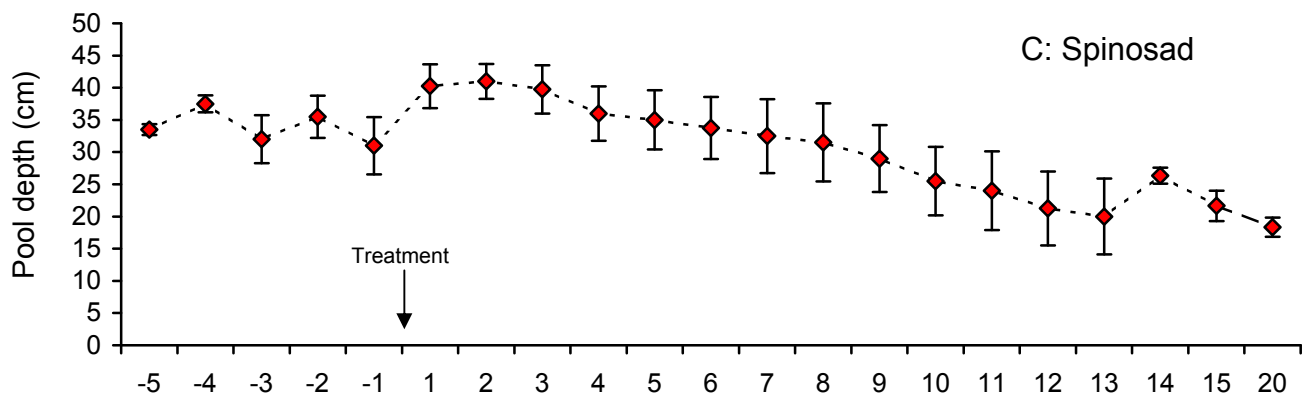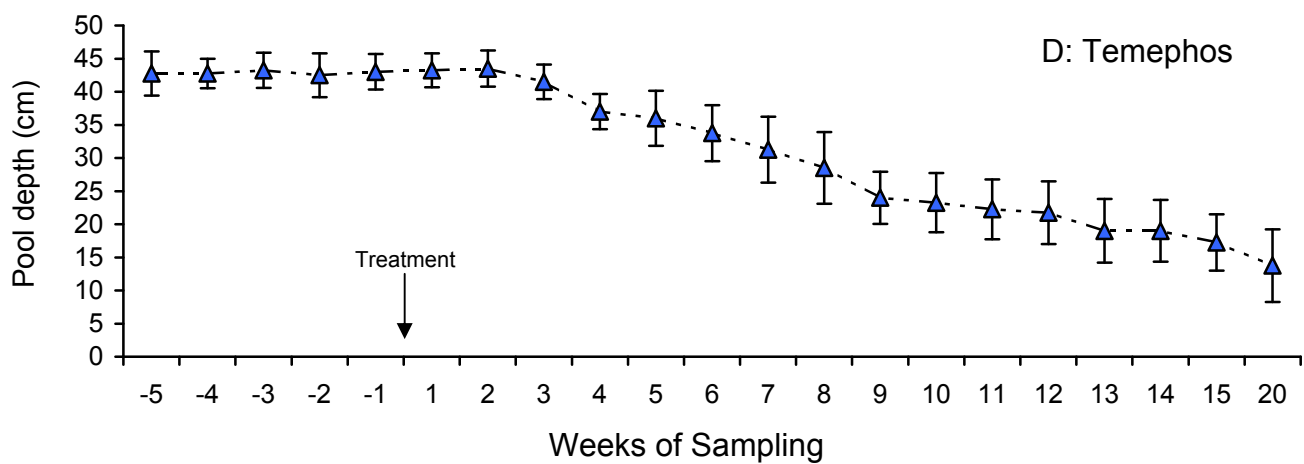

Supplement: Additional file 1: Figure S1 — Average water depth in experimental pools measured at moment of sampling during the period of the experiment. Pools were subjected to natural precipitation and water was not added by researchers at any time following construction and initial filling of the pools with water. At the final sample taken at 20 weeks post-treatment two pools in the spinosad treatment and one pool in the control treatment had dried up and were not included in the results. Vertical bars indicate SE. [file 1756-3305-7-55-S1.pdf]
